# Evaluating compulsory minimum volume standards in Germany: how many hospitals were compliant in 2004?

**DOI:** 10.1186/1472-6963-7-165

**Published:** 2007-10-17

**Authors:** Werner de Cruppé, Christian Ohmann, Karl Blum, Max Geraedts

**Affiliations:** 1 Professorship for Public Health, University Hospital of the Heinrich-Heine University Düsseldorf, Moorenstrasse 5, 40225 Düsseldorf, Germany; 2 Coordination Centre for Clinical Studies, University Hospital of the Heinrich-Heine University Düsseldorf, Moorenstrasse 5, 40225 Düsseldorf, Germany; 3 German Hospital Institute, Hansaallee 201, 40549 Düsseldorf, Germany

## Abstract

**Background:**

Minimum hospital procedure volumes are discussed as an instrument for quality assurance. In 2004 Germany introduced such annual minimum volumes nationwide on five surgical procedures: kidney, liver, stem cell transplantation, complex oesophageal, and pancreatic interventions. The present investigation is the first part of a study evaluating the effects of these minimum volumes on health care provision. Research questions address how many hospitals and cases were affected by minimum volume regulations in 2004, how affected hospitals were distributed according to minimum volumes, and how many hospitals within the 16 German states complied with the standards set for 2004.

**Methods:**

The evaluation is based on the mandatory hospital quality reports for 2004. In the reports, all hospitals are statutorily obliged to state the number of procedures performed for each minimum volume. The data were analyzed descriptively.

**Results:**

In 2004, 485 out of 1710 German hospitals providing acute care and approximately 0.14% of all hospital cases were affected by minimum volume regulations. Liver, kidney, and stem cell transplantation affected from 23 to hospitals; complex oesophageal and pancreatic interventions affected from 297 to 455 hospitals. The inter-state comparison of the average hospital care area demonstrates large differences between city states and large area states and the eastern and western German states ranging from a minimum 51 km^2 ^up to a maximum 23.200 km^2^, varying according to each procedure. A range of 9% – 16% of the transplantation hospitals did not comply with the standards affecting 1% – 2% of the patients whereas 29% and 18% of the hospitals treating complex oesophageal and pancreatic interventions failed the standards affecting 2% – 5% of the prevailing cases.

**Conclusion:**

In 2004, the newly introduced minimum volume regulations affected only up to a quarter of German acute care hospitals and few cases. However, excluding the hospitals not meeting the minimum volume standards from providing the respective procedures deserves considering two aspects: the hospital health care provision concepts by the German states as being responsible and from a patient perspective the geographically equal access to hospital care.

## Background

Volume-outcome associations have been broadly discussed since Luft's first investigations [1] in the late seventies. In the centre of attention are studies on single procedures, mostly complex surgical and other interventional procedures [2-11], and the intention to derive minimum volume standards. Methodological aspects have been debated as well [12-16] with particular focus on statistical adjustment procedures and on how minimum volumes effect the geographically equal access to care by causing centralisation of care [17-21] as well as accessibility of providers from the patients' perspective [22]. Review articles by Halm [23] and Gandjour et al. [24] resume and reflect the evidence. A summary by Shahian et al. [25] and an illustrative debate between Sheikh and Luft in Medical Care 2003 [26-28] depict the current state of debate.

Considering the potential of minimum volume standards to improve the quality of care, the German legislation decided in 2002 to use annual minimum volume standards as a quality assurance measure in the hospital sector. The German self administration in the health care sector discussed the procedures to be chosen and the respective thresholds. Following these discussions, the minimum volume regulation came into force in 2004 [29]. Five complex surgical procedures are consequently subjected to a minimum volume standard (Table 1): liver, kidney and stem cell transplantations, and complex oesophageal and pancreatic interventions. Hospitals which did not comply with the minimum volume standards in 2003 were no longer allowed to conduct these procedures in 2004.

**Table 1:** Annual minimum volume standards and their time of coming into effect in Germany

Minimum volume	MVS* suggested by health insurance funds	MVS since 2004	MVS since 2006
Liver transplantation	25/Hospital	10/Hospital	20/Hospital
Kidney transplantation	40/Hospital	20/Hospital	25/Hospital
Stem cell transplantation	20/Hospital	12 +- 2 [10–14]/Hospital	25/Hospital
Complex pancreatic intervention	10/Hospital	5/Hospital	10/Hospital
Complex oesophageal intervention	10/Hospital	5/Hospital	10/Hospital
Complex interventions of the mamma	150/Hospital	-	-
Heart transplantation	9/Hospital	-	-
Coronary surgery	100/Hospital	-	-
Carotis-TEA	20/Hospital	-	-
PCI	150/Hospital	-	-
Total knee replacement	-	-	50/Hospital**

* Minimum volume standard per year

** interim arrangement: hospitals with 40–49 total knee replacements and good quality can participate in 2006

Since the German self administration in the health care sector did not have comprehensive information on the current structure of hospital care with regards to the respective procedures, an evaluation study was funded to analyse the effects of the annual minimum volume standards on the structure and quality of hospital care in Germany. The following results are the first part of this study and focus on four questions:

1. How many hospitals performed minimum volume procedures in 2004?

2. How are the hospitals and cases affected by the minimum volume regulation distributed within the 16 German states (Lander) in 2004?

3. How many hospitals complied with the minimum volume standards in 2004?

4. How many cases are affected by hospitals which did not comply with the minimum volume standards in 2004?

As an introduction to the German context we first want to outline the background of the German minimum volume regulation, the German hospital landscape, and the available performance data of German hospitals in regard to minimum volumes.

### Minimum volumes in German hospitals

As mentioned above, beginning in 2004, minimum volume standards were implemented for the annual number of procedures performed for liver (≥ 10), kidney (≥ 20) and stem cell transplantations (≥ 10–14), and complex oesophageal (≥ 5) and pancreatic interventions (≥ 5). All minimum volume standards are valid for entire hospitals rather than for individual hospital departments. In addition, in 2004 and 2005 there was an annual minimum volume standard of five per surgeon for complex oesophageal and pancreatic interventions which were abandoned in 2006. Reasons will not be discussed in this particular study. The minimum volume regulation explicitly states exceptions such as emergency treatment, build up and renovation of a hospital or ensuring geographically equal access to care. The exact definition of each surgical procedure is done by means of the German Classification of Procedures in Medicine (OPS, Operationen- und Prozedurenschluessel). As a special feature of the minimum volume for liver transplantation it has to be mentioned that the OPS definition of the minimum volume for liver transplantation included in 2004 and 2005 so called "substitution" OPS procedures which were abolished in 2006. These entailed partial oncological liver resections and could be accounted for this minimum volume standard. The idea behind this decision was to allow hospitals performing liver transplantations to count comparable operations – which demonstrate their expertise on surgical liver procedures – on their annual minimum volume number. Hospitals that did not perform liver transplantations and therefore did not fall under this minimum volume regulation could however perform oncological liver operations which they were nonetheless obliged to document in their quality report for 2004. This distorts the corresponding number of hospitals performing liver transplantations and will be addressed where applicable.

The legal foundation for the German Healthcare System is found in Volume Five of the Social Legislation Code (Social Code Book No. 5, SCB V): Compulsory Health Insurance. A reform of the Social Code Book V in 2002 allowed German health care system's contracting parties to determine minimum volume standards on those planned services whose outcome quality is especially dependant on the performance volume. In 2003, the compulsory health insurance funds, as a contracting party, took the initiative to propose a list of ten minimum volumes [30] (Table 1). The first minimum volume regulation was finally adopted in December 2003 by the antecedent of the now responsible Federal Joint Committee (Gemeinsamer Bundesausschuss, G-BA), the primary decision-making body of the joint self-governing body of the German health care system since 2004. It comprises the National Association of Doctors and Dentists, the German Hospital Federation, and health insurance funds. National groups representing patients are given the right to file applications and to participate in the consultations of the Federal Joint Committee. The Federal Joint Committee updates the minimum volume regulation. The most recent sixth minimum volume for total knee replacement was introduced in 2006 with a minimum volume standard of 50 procedures per year.

### Hospitals in Germany

Hospital care in Germany is not organized by the Federal Administration but is rather a sovereign mandatory regulation of the 16 German states which form the Federal Republic of Germany. According to the Federal Statistical Office, there were 2166 hospitals in 2004 nationwide. Table 2 shows their distribution among the states. These hospitals provided 531,333 beds (equivalent to 6.4 beds per 1000 inhabitants) and treated 16.8 million cases with 8.7 days as the average length of stay [31]. In an international comparison, hospital care in Germany can be judged as an extensive and diverse inpatient service. Each state regulates hospital care by both a state hospital law and a state hospital plan specifying the regional distribution and size of each hospital including departments and number of beds provided. The plan is updated periodically assuring a need-oriented inpatient service with an equal distribution of hospitals state wide based on population density.

**Table 2:** The German states, size, population and hospitals in 2004

German states	surface* (km^2^)	inhabitants* (millions)	inhabitants*/km^2^	acute care hospitals with quality report	acute care hospitals with minimum volumes
Baden-Württemberg	35,752	10,7	299	212	54 (25%)
Bavaria	70,549	12,4	176	306	48 (16%)
Berlin	892	3,4	3811	53	26 (49%)
Brandenburg	29,478	2,6	88	49	11 (22%)
Bremen	404	0,66	1633	12	9 (75%)
Hamburg	755	1,7	2251	35	12 (34%)
Hesse	21,115	6,1	287	130	27 (21%)
Mecklenburg-Vorpommern	23,179	1,7	73	30	7 (23%)
Lower Saxony	47,620	8,0	168	173	41 (25%)
North Rhine-Westphalia	34,084	18,1	531	369	143 (24%)
Rhineland-Palatinate	19,854	4,1	207	91	22 (25%)
Saarland	2,569	1,1	428	25	6 (24%)
Saxony	18,415	4,3	233	75	27 (36%)
Saxony-Anhalt	20,446	2,5	122	45	15 (33%)
Schleswig-Holstein	15,763	2,8	178	61	17 (28%)
Thuringia	16,172	2.4	148	44	20 (45%)
Germany total	357,046	82,5	231	1710	485 (28%)

* [36]

Hospital case loads are negotiated on a local level between representatives of the hospital and those health insurance funds representing at least 5% of the hospital's patients. They agree upon an annual budget for the hospital following the last year's treatment cases, thus establishing case or procedure quantities only indirectly for the upcoming year. The annual budget is the principal activity in quantity control. Hospitals are classified into functional care levels defined by type and number of specialities and the total number of beds provided. The primary level of hospital care provision is comprised only of internal medicine and surgery departments. It is distinguished from the secondary level with additional departments such as gynaecology, ear, nose and throat department, paediatrics or neurology and from the maximum level with more than 15 specialities and more than 650 beds. When relating the hospital numbers of each care level to the total geographical size of Germany, an average care area of 250 km^2 ^results on the primary level with approximately 1400 hospitals. The medium level has a care area of 400 km^2 ^up to 1000 km^2^. The maximum level has a care area of 4000 km^2 ^with some 80 hospitals throughout Germany. There is no definition of geographically equal access to care, specifying the accessibility of the different hospital levels by an average distance or time. Only the state hospital plan of North Rhine Westphalia [32] defines as geographically equal access to care a maximum distance of 15 km for patients on the primary care hospital level in rural areas with unfavourable street connections. Otherwise it would be defined as 20 km to the next hospital. This corresponds to an average care area range of 700 km^2 ^to 1200 km^2^. It is preferred that patients attend a proximate hospital but they can still freely choose the hospital even outside their resident state and receive insurance coverage. Therefore it is important from a state perspective that patients (especially those living in a border area to another state) use the hospital of the neighbouring state. This is especially relevant for the three city states Berlin, Bremen and Hamburg. These provide hospital care for a part of the neighbouring state's population since as metropolis and sovereign states they are equipped with a better infrastructure than the areas of the bordering state. The hospital care rate of each state expresses this difference between resident and non-resident patients treated in that particular state. In 2004 this care rate was 145% for Bremen (i.e. 45% of the treated patients originated from the Bremen inclusive state of Lower Saxony). The care rate for Hamburg was 124% due to patients from the neighbouring states of Lower Saxony and Schleswig-Holstein. For Berlin, the care rate was 111% including patients from Brandenburg which itself has a rate of 88% (i.e. 12% of its patients were treated outside the state). All other states have a care rate of 94% to 105% [based on a personal inquiry from the Federal Statistical Office Germany].

### Hospital performance data

German hospitals are obliged by law to convey their performance data primarily to five official authorities. First, some global hospital data (e.g. cases, age, sex, length of stay) are reported to the responsible statistical state office. Secondly, they report to the regional health insurance funds basic hospital case data (e.g. ICD, ICPM, discharge diagnosis, age, sex) but only on their respective insurees. These data are not publicly available. Thirdly, hospitals are required to document process and outcome data for the legally obligatory external quality assurance, which covered some 30 procedures in 2004 (mostly surgical interventions) and about 15% of all hospital cases [33]. These data are nationwide aggregated and published anonymously . In 2004 the external quality assurance did not cover any data on procedures that fell under the minimum volume regulation. Fourthly, with the introduction of the DRG Reimbursement System all relevant data on patients, diagnostic and treatment procedures are conveyed to the Institute for the Hospital Reimbursement System (InEK). This institute was founded in 2003 and uses the data to develop the G-DRG-System (German Diagnosis Related Groups). These data are published annually on a federal aggregated level, limiting in depth analyses.

It became obvious that following the self-governing bodies' decision in 2003 on the minimum volume regulation, there was no comprehensive, systematic, and publicly accessible data available on the number and distribution of hospitals performing relevant procedures, their cases and performance affected by possible minimum volume standards.

In the meantime, a fifth source of information has been established. Beginning in 2004, all German hospitals are obliged to publish a structured quality report biannually covering the previous year [34]. The hospitals provide the reports to the health insurance funds which then publish them on the internet. The hospital quality reports for 2004 were published for the first time in August 2005. They are legally based on the list of quality assurance instruments in the Social Code Book, where these reports were introduced in 2003 along with minimum volumes in 2002. As part of these reports, each hospital is required to give detailed information on those procedures for which minimum volume standards have been defined. Therefore this data source is currently the only available information on all procedures that are covered by minimum volume regulation.

## Methods

### Data source

Our data is based on all German hospital quality reports for 2004 published by the Association of Health Insurance Funds by December 2005. These reports are freely accessible via http://www.g-qb.de.

### Data analysis and validation

The quality reports were analysed according to hospital type, hospital location, and information on type and number of procedures with minimum volume standards conducted (including the OPS number level).

In the first step we validated type and location of each hospital by comparing them with the hospital information system of the German Hospital Institute. This was necessary to remove duplicate reports and include only hospitals which work in the acute care setting. Psychiatric and neurological units without a neurological acute care unit, geriatric and rehabilitative units without acute care, palliative medical care units and special hospitals for addiction were all excluded.

We performed descriptive statistical procedures to analyze the hospital data by using the statistical programme SPSS.

Hospitals and case numbers on liver and kidney transplantation were validated by the information given in the 2004 report of the German Foundation for Organ Transplantation (DSO) http://www.dso.de[35]. Since stem cells are not defined as organs under the German transplantation law there is no corresponding data available in the DSO report.

### Study sample

There were 1810 hospitals authorized for acute care in 2004. By December 2005, 1710 of these hospitals had published a quality report with information on procedures covered by the minimum volume regulation which is the reference group of the following analysis.

### Hospital care density as a specific indicator

This investigation uses the proxy "hospital care density" as an indicator of accessibility. This is defined as the mean geographical surface area (in km^2^) a hospital serves. The indicator is calculated by dividing the state or federal surface area by the number of hospitals serving one of the minimum volume procedures. It is used as an indicator for the average hospital care area and hospital accessibility.

## Results

The 1710 quality reports show that 485 hospitals (i.e. 28% of all German hospitals), are affected by at least one minimum volume in 2004. This proportion varies from 16% in Bavaria up to 75% in Bremen (Table 2). The five minimum volumes in 2004 affected 23,128 cases which comprise 0.14% of the 16.8 million cases treated in German hospitals that year. Out of all 23,128 minimum volume cases 736 cases (3%) were treated in hospitals not complying with the required minimum volume standard. Detailed information on hospital numbers, cases, and hospital care area per state and minimum volume are given in the following results.

### Liver transplantation

According to the quality reports, those procedures belonging to the minimum volume for liver transplantations were conducted by 132 hospitals with 3703 cases (Table 3). The average care area of a hospital was 2704 km^2 ^varying from a minimum of 149 km^2 ^in Berlin to a maximum of 11905 km^2 ^in Lower-Saxony. Throughout Germany 70 hospitals (53%) complied with the minimum volume standard of 10 interventions per hospital per year. Considering the influence of the aforementioned "substitution" OPS procedures (oncological liver operations) on this minimum volume standard and focusing the evaluation only on hospitals performing actual liver transplantation OPS procedures, only 23 hospitals conducted liver transplantations. This could be completely validated by the DSO report confirming these 23 hospitals as the actual liver transplantation centres in Germany which all fulfilled the minimum volume standard.

**Table 3:** Hospitals performing the minimum volume regulation liver transplantation and official number of liver transplantation centres in 2004

German states	MV* hospitals	km^2^/MV hospitals	MV hospitals, standard achieved		Number of cases in MV hospitals with standard NOT achieved		Transplan-tation centres (DSO**)
Baden-Württemberg	20	1788	11	55%	34	7%	2
Bavaria	13	5427	9	69%	24	5%	5
Berlin	6	149	3	50%	18	4%	1
Brandenburg	3	9826	1	33%	8	26%	0
Bremen	2	202	2	100%	0	0%	0
Hamburg	4	189	3	75%	1	0%	1
Hesse	5	4223	4	80%	9	6%	1
Mecklenburg-Vorpommern	3	7726	2	67%	6	12%	1
Lower Saxony	4	11905	2	50%	4	3%	2
North Rhine-Westphalia	51	668	22	43%	100	12%	4
Rhineland-Palatinate	2	9927	1	50%	3	2%	1
Saarland	2	1285	1	50%	5	5%	1
Saxony	8	2302	4	50%	8	7%	1
Saxony-Anhalt	2	11223	1	50%	2	2%	1
Schleswig-Holstein	4	3941	3	75%	3	3%	1
Thuringia	3	5391	1	33%	16	14%	1
Germany total	132	2.704	70	53%	241	7%	23

* Minimum volume

** German foundation for organ transplantation

### Kidney transplantation

OPS minimum volume procedures for kidney transplantation were conducted by 43 hospitals with 2489 cases treated. There were 39 hospitals (91%) which complied with the required minimum volume of 20 interventions. Hospitals not meeting the standard treated 2% of the cases. The DSO report names 40 kidney transplantation centres (Table 4) where 2320 transplantations were performed. Among those 40 centres, two hospitals located in one city (Berlin and Cologne) were subsumed twice as one centre while the quality reports counted them separately. Therefore both data sources differ in the way that only one hospital conducted one special kidney surgery according to its OPS without being a kidney transplantation centre. Hence both reports are congruent. The average care area per hospital was 8303 km^2^, varying from 404 km^2 ^in Bremen to 23179 km^2 ^in Mecklenburg-Vorpommern.

**Table 4:** Hospitals performing the minimum volume regulation kidney transplantation and official number of kidney transplantation centres in 2004

German states	MV* hospitals	km^2^/MV hospitals	MV hospitals, standard achieved		Number of cases in MV hospitals with standard NOT achieved		Transplantation centres (DSO**)
Baden-Württemberg	6	5959	6	100%	0	0%	6
Bavaria	6	11758	5	83%	18	5%	6
Berlin	3	297	3	100%	0	0%	2
Brandenburg	0		0				0
Bremen	1	**404**	1	100%	0	0%	1
Hamburg	1	755	1	100%	0	0%	1
Hesse	4	5279	3	75%	10	6%	4
Mecklenburg-Vorpommern	1	**23179**	1	100%	0	0%	1
Lower Saxony	3	15873	2	67%	10	3%	3
North Rhine-Westphalia	9	3787	8	89%	1	0%	7
Rhineland-Palatinate	2	9927	2	100%	0	0%	2
Saarland	1	2569	1	100%	0	0%	1
Saxony	2	9028	2	100%	0	0%	2
Saxony-Anhalt	1	20446	1	100%	0	0%	1
Schleswig-Holstein	2	7882	2	100%	0	0%	2
Thuringia	1	16172	1	100%	0	0%	1
Germany total	43	8303	39	91%	39	2%	40

* Minimum volume

** German foundation for organ transplantation

### Stem cell transplantation

OPS minimum volume procedures for stem cell transplantation were conducted by 82 hospitals. There were 5178 cases where 69 hospitals (84%) complied with the minimum volume standard of 12 interventions. 16% of the hospitals did not meet the requirement affecting 0.15% of the cases. The average care area per hospital was 4354 km^2^, which varied from 202 km^2 ^in Bremen to 16172 km^2 ^in Thuringia (Table 5).

**Table 5:** Hospitals performing the minimum volume regulation stem cell transplantation

German states	MV* hospitals	km^2^/MV hospitals	MV hospitals, standard achieved		Number of cases in MV hospitals with standard NOT achieved	
Baden-Württemberg	13	2750	8	62%	41	4%
Bavaria	8	8819	7	88%	1	0%
Berlin	4	223	4	100%	0	0%
Brandenburg	4	7370	3	75%	2	3%
Bremen	2	**202**	2	100%	0	0%
Hamburg	3	252	3	100%	0	0%
Hesse	8	2639	5	63%	11	4%
Mecklenburg-Vorpommern	2	11590	2	100%	0	0%
Lower Saxony	5	9524	5	100%	0	0%
North Rhine-Westphalia	19	1794	16	84%	22	2%
Rhineland-Palatinate	3	6618	3	100%	0	0%
Saarland	1	2569	1	100%	0	0%
Saxony	2	9208	3	100%	0	0%
Saxony-Anhalt	4	5112	3	100%	0	0%
Schleswig-Holstein	3	5254	3	100%	0	0%
Thuringia	1	**16172**	1	100%	0	0%
Germany total	82	4.354	69	84%	77	1%

* Minimum volume

### Complex oesophageal interventions

OPS minimum volume procedures for complex oesophageal interventions were conducted by 297 hospitals. There were 3302 cases where 211 hospitals (71%) met the standard of five interventions. Those 29% of hospitals not meeting the standard affected 5% of the cases and were mainly located in the large-area states (Table 6). The average care area per hospital was 785 km, which varied from 51 km^2 ^in Bremen to 3311 km^2 ^in Mecklenburg-Vorpommern.

**Table 6:** Hospitals performing the minimum volume regulation complex oesophageal intervention

German states	MV* hospitals	km^2^/MV hospitals	MV hospitals, standard achieved		Number of cases in MV hospitals with standard NOT achieved	
Baden-Württemberg	35	1021	23	66%	28	7%
Bavaria	28	2519	20	71%	14	3%
Berlin	17	52	12	71%	8	4%
Brandenburg	7	**4211**	5	71%	5	8%
Bremen	8	**51**	7	88%	3	4%
Hamburg	7	108	7	100%	0	0%
Hesse	16	1320	13	81%	6	3%
Mecklenburg-Vorpommern	6	3863	6	100%	0	0%
Lower Saxony	27	1764	20	74%	20	7%
North Rhine-Westphalia	93	367	64	69%	52	7%
Rhineland-Palatinate	11	1805	8	73%	4	3%
Saarland	2	1285	1	50%	4	10%
Saxony	12	1535	8	67%	14	11%
Saxony-Anhalt	9	2272	6	67%	3	2%
Schleswig-Holstein	12	1314	8	67%	9	9%
Thuringia	7	2310	3	43%	9	33%
Germany total	297	1.202	211	71%	179	5%

* Minimum volume

### Complex pancreatic interventions

OPS minimum volume procedures for complex pancreatic interventions were conducted by 455 hospitals. There were 8417 cases where 373 hospitals (82%) complied with the standard of five interventions. Those 18% which did not comply affected 2% of the cases. In Saxony, Saxony-Anhalt, North Rhine-Westphalia, and Berlin, some 30% did not meet the standard. The average care area per hospital was 1202 km^2^, including 51 km^2 ^in Bremen and 4211 km^2 ^in Brandenburg (Table 7).

**Table 7:** Hospitals performing the minimum volume regulation complex pancreatic intervention

German states	MV* hospitals	km^2^/MV hospitals	MV hospitals, standard achieved		Number of cases in MV hospitals with standard NOT achieved	
Baden-Württemberg	49	730	42	86%	15	1%
Bavaria	46	1534	43	93%	8	1%
Berlin	25	**36**	18	72%	21	3%
Brandenburg	11	2680	11	100%	0	0%
Bremen	8	51	8	100%	0	0%
Hamburg	10	76	9	90%	4	1%
Hesse	27	782	23	85%	7	1%
Mecklenburg-Vorpommern	7	**3311**	7	100%	0	0%
Lower Saxony	38	1253	32	84%	17	3%
North Rhine-Westphalia	134	254	102	76%	76	4%
Rhineland-Palatinate	21	945	14	67%	14	6%
Saarland	5	514	4	80%	2	2%
Saxony	21	877	15	71%	17	4%
Saxony-Anhalt	18	1136	13	72%	13	5%
Schleswig-Holstein	16	985	15	94%	1	0%
Thuringia	19	851	17	89%	5	2%
Germany total	455	785	373	82%	200	2%

* Minimum volume

## Discussion

In 2004, the introduction of five minimum volumes in Germany affected 28% of all hospitals delivering these procedures, reflecting a minimum of 16% and a maximum of 75% of hospitals on the state level and 0.14% of all hospital cases. The city states show a higher hospital care density than the large-area states. Among the latter a higher density of hospital care can be seen in the Western states as opposed to the Eastern ones. The number of affected hospitals also depends on the degree of intervention specialisation. Liver, kidney and stem cell transplantations are highly specialised treatments and with only 23 to 82 delivering hospitals they are already centralised. The complex oesophageal and pancreatic interventions however affect the medium hospital care level of which 18% and 29% respectively did not comply with the standards set in 2004. Their withdrawal from care provision as a result of not meeting the minimum volume standards might entail a stronger regionalisation. The number of cases treated by these hospitals shows that with the minimum volume standard of five interventions per year in 2004 however, only 3% of the cases were to be relocated.

German federal law introduced in 2002 the possibility of setting minimum volume standards by the joint self-governing body of the German Health Care System with the explicit intention to improve hospital quality of care. The joint self-governing body of the German Health Care System gave therefore a rationale in late 2003 for the introduction beginning in 2004 of minimum volume standards on five surgical interventions. The effects on the hospital level, the case level, and geographically access to care for the German hospital landscape were not assessed scientifically beforehand for one primary reason. At that time there was no comprehensive nationwide hospital performance data available except from a dozen specific surgical interventions (where no minimum volume standard was applied) documented in the obligatory external quality assurance measure. In fact, hospital quality reports are the first extensive, comparable and accessible data source detailing Germany's hospital performance on the introduced minimum volume standards but available only since 2004, the same year these minimum volume standards were introduced. However, these reports do not include data on the quality of care related to the minimum volume standards. Therefore there is no opportunity for a longitudinal analysis, i.e. to compare hospital care provision data from before and after the introduction of these minimum volume standards, neither regarding the structure of care provision or their quality. Since our evaluation study starts with the description of how many hospitals and cases got affected in 2004, the first year the annual minimum volume standards came into effect in German hospitals, the study can not quantify the number of hospitals which stopped performing the procedures by the end of 2003 because they assumed not being able to fulfil the annual standards set for 2004.

The first result of this study is that in 2004 485 hospitals representing 28% of all German hospitals were impacted by the quality assurance instrument minimum volume standard. The rather high degree of specialisation and the low required case number of the introduced annual minimum volume standards, on which the joint self-governing body agreed upon however, had the effect that these five minimum volumes cover only 23.128 cases representing 0.14% of all hospital cases. But both effects, the number of hospitals and cases affected, differ between the five respective surgical interventions depending on their degree of specialisation and the existing hospital care structure in the 16 German states.

We have chosen the average area for which a hospital has to provide care, called here "hospital care density", as an indicator and a proxy for accessibility of a hospital. We are aware of the limitation this mere surface parameter implies for the relevant patient information on how far the distance is to a hospital either in km or travel time. Due to the available data this important information can not yet be presented.

On the federal level the average area for a hospital providing liver and kidney transplantation is 15.500 km^2 ^and 8300 km^2 ^respectively and exceeds even the mean area a hospital serves on the maximum hospital (comprising more than 15 specialities and more than 650 beds) level which is approximately 4000 km^2 ^on average. This demonstrates the already highly centralised provision of care in these chosen minimum volume procedures. Stem cell transplantations meet this maximum hospital level with a density of 4350 km^2^. In contrast, the number of hospitals conducting complex oesophageal (297) and complex pancreatic (455) procedures is equivalent to the secondary hospital level, i.e. district level, of approximately 1000 km^2^. In these cases the minimum volume standards affect far less centralised procedures. On a state level the area size for all five minimum volumes varies considerably from 50 km^2 ^to 400 km^2 ^in the city states. The largest areas covered by the hospitals in the eastern German states vary from 3300 km^2 ^to 23.000 km^2^. This asymmetry must be viewed taking into consideration the urban rural gap with very different population densities as well as the fact that the city states provide care for part of the population of bordering states.

How many hospitals actually complied with the standards set for 2004? All 23 liver transplantation centres in Germany met the standard, as did 38 of the 42 kidney transplantation centres and 84% of the stem cell transplantation hospitals. The highest failure rates with 29% and 18% affect complex oesophageal and pancreatic interventions. All hospitals met the standard in only two and three states respectively. It should be noted that most of the city states' large hospital size as well as the already more centralised hospital structure of the eastern states helped enable standards to be met. Non-compliant hospitals are located especially in the large area states of the western part with a quarter to a third being affected. The defined standard of five interventions for 2004 results in only about 5% and 1% of the cases being affected by a potential withdrawal of these hospitals. This reduces the number of providing hospitals considerably but with presumably only a slight effect on the overall population. However the standards for 2006 have been doubled for all minimum volumes with the exception of kidney transplantation. This might result not only in the withdrawal of more hospitals from providing care but also in affecting many more patients and hence having a stronger impact on the accessibility of hospital care on a population level in most parts of Germany, at least for the concerned interventions. This will be seen in the second series of hospital quality reports for 2006 being published by the end of 2007.

These first basic results on minimum volume effects on the hospital landscape in Germany highlight state and patient perspectives. The minimum volumes have been introduced on a federal level but the states are ultimately responsible for providing the hospital care structure with the obligation to consider the need and accessibility of its population state wide. The patients might have to face a reduction in the range of providers when hospitals will not offer a procedure due to unmet standards and they might have to deal with a different and perhaps longer route to the proximate hospital. But these detailed aspects of patient interest cannot be answered with the available data.

The states' aspect is twofold. On one side, an increasing regionalisation or centralisation on the maximum hospital level will encourage at least some states to cooperate with neighbouring states in providing high performance treatments such as transplantations to its population. Today all states have at least one transplantation centre for each of the three types of transplantation with minimum volumes with the exception of Brandenburg, which lacks a kidney transplantation centre. A process of centralisation will challenge the states' autonomy and request cooperation. On the other side, centralisation on the medium or primary hospital level concerns every state with regard to planning and managing the hospital care provision in its own realm. This will challenge the states to adjust their state hospital plan and develop a manageable definition of geographically equal access to care while balancing patient needs for reasonable accessibility of hospital care and minimum volume regulation requirements. This balance will most likely be different for each type of medical intervention considering the disease prevalence, the degree of treatment specialisation, and the distributable financial resources. The question of how closely the 16 states will work together on these forthcoming challenges remains open. If different concepts will be applied it might be worthwhile to consider a benchmarking system which compares the different approaches in the states and might help learning from each other.

It has to be stated critically that the hospital quality reports could not have been validated and proofed for comprehensiveness. The partial validation by the 2004 report of the German Foundation for Organ Transplantation however, indicated a good validity for liver and kidney transplantation. Further validation of these data will only be possible when primary and further secondary data sources become accessible in the course of the ongoing evaluation.

## Conclusion

The nationwide introduction of the first five annual minimum volumes for German hospitals in 2004 seems to be of modest effect on the already highly specialised transplantation treatments. It appears to have moderate effect on the provision of oesophageal and pancreatic surgery, thereby reducing the number of small providers without affecting large populations. But it has to be stated that these conclusions can be drawn only for 2004 and do not take hospitals into consideration which withdrew or added a procedure by the end of 2003 due to the introduction of minimum volume standards. The considerably heightened standards, already in effect since 2006, will most likely raise the scope of minimum volume effects from what could now be seen as a process consolidation of reallocating the hospital care provision in most parts of Germany. This might be enhanced by successively distending minimum hospital volumes on a greater number of interventions as already happened in 2006 by introducing minimum volume standards on knee-replacement procedures (50 per year) and on cardiac surgery (still without a number agreed upon). This process will challenge the accessibility of hospital care for patients. It will additionally challenge the interstate cooperation on high performance medicine of the maximum hospital care level and the development of care concepts within each state on the primary and secondary hospital care level. This could result in avoidance of side effects from potential centralisation tendencies.

## Competing interests

The author(s) declare that they have no competing interests.

## Authors' contributions

WDC conceived and designed the study, performed data analysis, drafted manuscript. MG conceived and designed the study, performed data analysis, and revised the manuscript critically for important intellectual content. CO has contributed to conception and design of the study, assisted with data analysis, and revised the manuscript critically for important intellectual content. KB has contributed to conception and design of the study, and revising the manuscript critically for important intellectual content. All authors have read and approved the final version of the paper.

## Pre-publication history

The pre-publication history for this paper can be accessed here:


